# Anti-HPV gel versus interferon α-2b gel as adjuvant therapy after LEEP for HSIL with HR-HPV infection on clinical outcomes, vaginal microecology, and biomarkers of proliferation and immunity

**DOI:** 10.3389/fonc.2026.1784137

**Published:** 2026-03-25

**Authors:** Xu Long, Mengjun Luo, Chunrong Yang, Fengying Yan, Hongou Pu, Xiaohong Li

**Affiliations:** 1 Affiliated Women’s and Children’s Hospital of the University of Electronic Science and Technology of China, Gynecology Department of Chengdu Women’s and Children’s Central Hospital, Chengdu, Sichuan, China; 2 Clinical Laboratory Department, Women’s and Children’s Hospital Affiliated to University of Electronic Science and Technology of China, Chengdu Women’s and Children’s Central Hospital, Chengdu, Sichuan, China; 3 Zhongyi Hospital of Traditional Chinese Medicine, Department of Gynecology, Chengdu, Sichuan, China; 4 Sichuan Academy of Medical Sciences, Sichuan Provincial People’s Hospital, Department of Gynecology, Chengdu, Sichuan, China; 5 Affiliated Women’s and Children’s Hospital of the School of Medicine, University of Electronic Science and Technology of China, Chengdu Women’s and Children’s Central Hospital Traditional Chinese Medicine Gynecology, Chengdu, Sichuan, China

**Keywords:** anti-HPV gel, high-grade squamous intraepithelial lesion, high-risk human papillomavirus, interferon α-2b gel, Ki-67, loop electrosurgical excision procedure, survivin, vaginal microecology

## Abstract

**Objective:**

To evaluate and compare the efficacy, impact on vaginal microbiota, changes in inflammatory/immune/proliferation biomarkers, and safety profile of Anti-HPV gel versus Interferon α-2b gel as adjuvant therapies after LEEP in patients with HSIL and HR-HPV infection.

**Methods:**

This retrospective cohort study included 207 eligible patients diagnosed with HSIL and HR-HPV infection who underwent initial LEEP between January 2023 and January 2025. Patients were divided into a control group (n=102, Interferon α-2b gel) and an observation group (n=105, Anti-HPV gel), both receiving two standardized treatment courses post-operatively. Outcome measures included total clinical effective rate, vaginal microecological recovery (pH, Nugent score), serum inflammatory cytokines (IL-17, TNF-α, IFN-γ, TGF-β), immunoglobulins (IgA, IgG, IgM), cell proliferation markers (serum Survivin, cervical tissue Ki-67), adverse events, and 6-month HR-HPV recurrence rate. An exploratory model was constructed using post-treatment indicators to predict recurrence.

**Results:**

Compared with the control group, the observation group showed superior outcomes across multiple domains: a higher total clinical effective rate (87.62% vs. 75.49%, P = 0.024); better restoration of vaginal microecology with lower post-treatment pH (4.29 ± 0.37 vs. 4.78 ± 0.42, P<0.001) and Nugent score (2.96 ± 1.15 vs. 4.52 ± 1.37, P<0.001); greater reductions in serum inflammatory markers (IL-17, TNF-α, IFN-γ, TGF-β, all P<0.001); greater increases in immunoglobulins (IgA, IgG, IgM, all P<0.001); more pronounced decreases in cell proliferation markers (serum Survivin and tissue Ki-67, both P<0.001); a lower 6-month HR-HPV recurrence rate (6.67% vs. 16.67%, P = 0.024) with comparable overall adverse event incidence (10.48% vs. 13.73%, P = 0.473). The exploratory predictive model based on post-treatment indicators demonstrated good discrimination for recurrence (AUC = 0.812, 95% CI: 0.747–0.878).

**Conclusion:**

In conclusion, as a postoperative adjuvant, Anti-HPV gel demonstrated superior clinical and biomarker outcomes compared to Interferon α-2b gel, with a favorable safety profile and lower short-term recurrence risk, suggesting its promising clinical value pending prospective confirmation.

## 1 Introduction

High-grade squamous intraepithelial lesion (HSIL) is a critical precancerous stage in the development of cervical cancer, with a significantly higher risk of progression than low-grade lesions. If not treated in a timely and effective manner, some patients may further develop invasive cervical cancer (1). A large body of evidence (2–4) has demonstrated that persistent infection with high-risk human papillomavirus (HR-HPV) is the core etiological factor driving the occurrence and progression of HSIL. Continuous viral replication, immune evasion, and the virus-induced chronic inflammatory microenvironment play important roles in lesion evolution. Therefore, how to promote HPV clearance, improve the local microenvironment, and reduce the risk of recurrence while removing the lesion has become a key issue in the clinical management of HSIL. Loop electrosurgical excision procedure (LEEP), as a standard surgical treatment for HSIL, has been widely used in clinical practice due to its advantages of simple operation, thorough lesion removal, and relatively limited tissue damage. However, LEEP primarily targets visible lesions and has limited effects on underlying persistent HPV infection and postoperative local immune imbalance. Consequently, some patients may still experience persistent HR-HPV positivity, vaginal microecological disturbances, and lesion recurrence after surgery (5, 6). Therefore, the combination of appropriate local adjuvant therapy after LEEP to enhance antiviral capacity, regulate immune responses, and improve the microecological environment has gradually become a focus of clinical research and practice.

Interferon α-2b is a cytokine with broad antiviral, immunomodulatory, and antiproliferative effects. By enhancing immune responses, inhibiting viral replication, and inducing apoptosis of abnormal cells, it plays a certain role in the treatment of HPV-related diseases (7, 8). When formulated as a gel, interferon α-2b can act directly on the cervical area, to some extent improving local immune status and promoting HPV clearance. However, some studies (9) have shown that interferon α-2b still has certain limitations in terms of HPV clearance rates and long-term recurrence control, with its efficacy being greatly influenced by individual immune status and the local microenvironment. In recent years, anti-HPV gel, as a novel local therapeutic preparation, has gradually been applied in clinical practice. Such preparations exert their effects through multiple mechanisms, including the formation of a physical barrier, interference with viral adsorption and invasion, modulation of the local mucosal environment, and promotion of tissue repair. They have shown potential in improving vaginal and cervical microecology and promoting HPV clearance (10). Previous studies (11) have suggested that anti-HPV gel may have favorable safety and application prospects as an adjuvant treatment for HPV infection–related cervical lesions; however, its comprehensive effects in HSIL patients after LEEP remain to be systematically evaluated.

Based on the above background, the present study adopted a retrospective cohort design to analyze patients with HSIL complicated by HR-HPV infection who underwent LEEP at our hospital. By comparing two postoperative adjuvant treatment regimens—interferon α-2b gel and anti-HPV gel—the study evaluated differences in clinical efficacy, vaginal microecology, inflammatory and immune indicators, and the expression of cell proliferation–related biomarkers, as well as short-term recurrence and safety during follow-up, aiming to generate comparative evidence that could guide the selection of optimal adjuvant therapy following LEEP for HSIL patients with HR-HPV infection.

## 2 Data and methods

### 2.1 Study subjects

**Figure 1:**
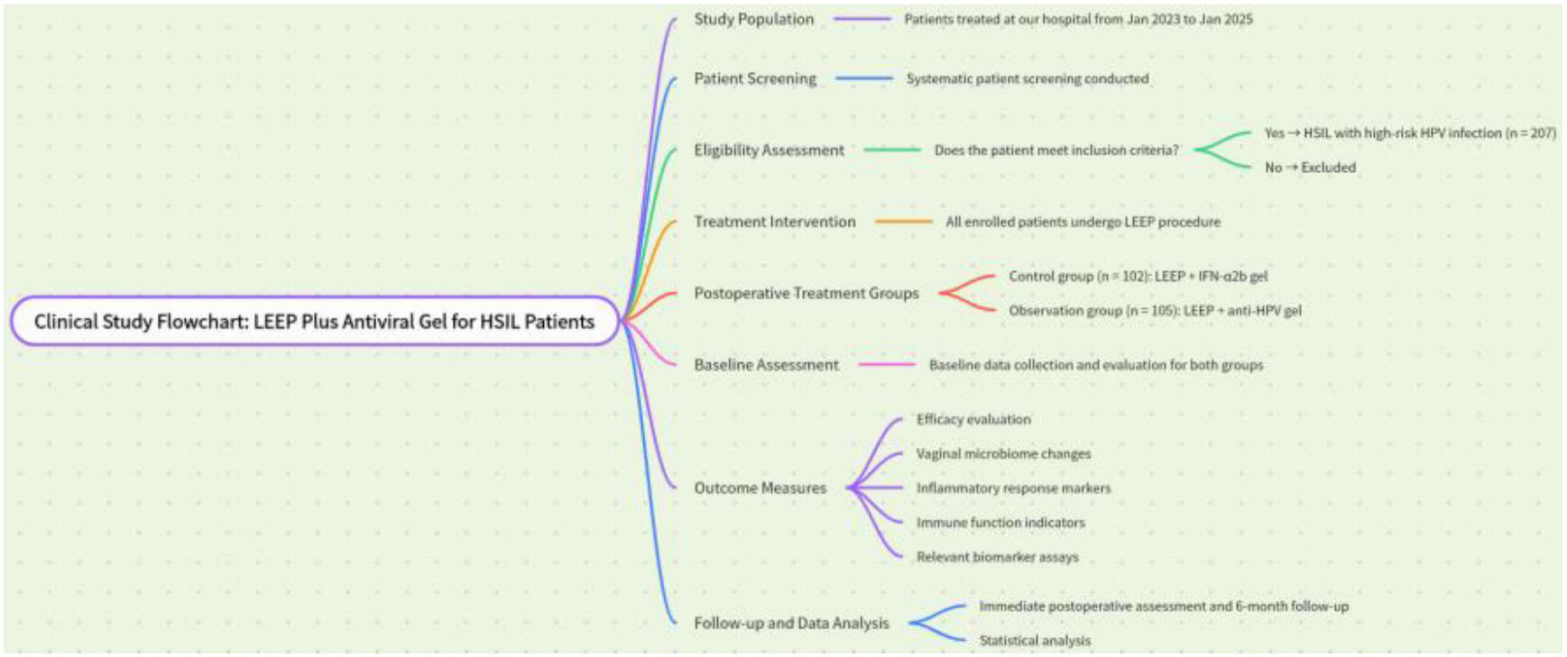
Flowchart of patient enrollment, treatment, and follow-up.

This study adopted a retrospective cohort design. Patients who attended the Department of Gynecology of our hospital between January 2023 and January 2025, were pathologically diagnosed with HSIL, had documented HR-HPV infection at baseline (defined as the most recent HR-HPV test result before initiation of postoperative adjuvant therapy), and underwent LEEP were selected as the study subjects. For patients with infection by two or more HR-HPV subtypes, the predominant genotype reported by the assay (e.g., the highest viral load or signal intensity, depending on assay output) was recorded as the primary genotype for distribution analyses, and the number of subtypes (single vs. multiple) was recorded separately. A total of 207 patients who met the study criteria were included. The sample size was determined based on the actual number of HSIL patients with HR-HPV infection who met the inclusion and exclusion criteria within the study period, and no *a priori* sample size calculation was performed. All included patients underwent LEEP for the first time, and complete clinical data were available, including general demographic information, pathological findings, laboratory test results, and follow-up records. Study subjects were strictly screened according to predefined inclusion and exclusion criteria to minimize the impact of selection bias on the study results. A brief overview of the study flow is shown in **Figure 1** below.

### 2.2 Inclusion and exclusion criteria

Inclusion criteria: (1) HSIL confirmed by cervical biopsy or postoperative pathological examination after LEEP; (2) HR-HPV infection confirmed by a documented HR-HPV test prior to initiation of postoperative adjuvant therapy (preferably preoperatively; if a preoperative report was not available in the medical record, the earliest postoperative HR-HPV test performed before starting adjuvant therapy was used as the baseline confirmation); (3) met the indications for LEEP and received LEEP at our hospital, with postoperative adjuvant treatment using either interferon α-2b gel or anti-HPV gel; (4) age 18–60 years; (5) complete clinical and follow-up data available for efficacy and safety evaluation.

Exclusion criteria: (1) concomitant invasive cervical cancer or other gynecological malignancies; (2) previous cervical conization, LEEP, or other surgical treatment for HSIL; (3) concomitant severe immune system diseases, long-term use of immunosuppressants or glucocorticoids; (4) pregnancy or lactation; (5) concomitant severe hepatic, renal, or cardiac dysfunction, or other systemic diseases that could affect the evaluation of study indicators; (6) missing follow-up data or inability to complete 6 months of follow-up.

### 2.3 Ethical statement

This study was a single-center retrospective study, and all study subjects were derived from previous clinical diagnostic and treatment data, without additional interventions or new risks to patients. The study protocol was reviewed and approved by the Medical Ethics Committee of our hospital (Ethics approval No.: 24FKLC-YWDB09) and was conducted in strict accordance with the principles of the Declaration of Helsinki. During data collection and analysis, patient personal information was anonymized and used solely for scientific research purposes to fully protect patient privacy and data security. Given the retrospective design of this study and the absence of additional diagnostic or therapeutic interventions, informed consent was waived with the approval of the Ethics Committee.

### 2.4 Data retrieval and extraction process

Data for this retrospective cohort study were systematically extracted from the hospital’s integrated electronic health record system. The retrieval process was conducted in a structured, multi-step manner to ensure completeness and accuracy. First, a preliminary list of all patients who underwent loop electrosurgical excision procedure between January 1, 2023, and January 31, 2025 was generated using the relevant procedural codes. This initial cohort was then screened against pre-defined inclusion and exclusion criteria. For each eligible patient, a standardized electronic case report form was used to extract the following variables: demographic characteristics; preoperative and postoperative histopathology reports; high-risk HPV genotyping results (tested using cobas^®^ 4800 HPV Test); details of the adjuvant therapy prescribed (including product name, manufacturer, dosage, and treatment schedule); vaginal microenvironment indices (pH, Nugent score, lactobacillus status); laboratory results for inflammatory cytokines (IL-17, TNF-α, IFN-γ, TGF-β), immunoglobulins (IgA, IgG, IgM), and serum survivin; immunohistochemistry records for tissue Ki-67 expression; and follow-up records including HPV retesting results and documentation of any adverse events. Two trained research assistants independently performed the data extraction. Any discrepancies were resolved through discussion and, when necessary, by adjudication from a senior investigator. All extracted data were anonymized and securely stored in a password-protected database for subsequent analysis.

#### 2.4.1 Laboratory assay specifications

The specific assays, reagents, and key procedural details are provided as follows.

##### 2.4.1.1 HR-HPV genotyping

The presence and genotyping of high-risk HPV were determined using the cobas^®^ 4800 HPV Test (Roche Molecular Systems, Pleasanton, CA, USA) on cervical exfoliated cell samples, following the manufacturer’s protocol. This fully automated, real-time PCR assay qualitatively detects 14 high-risk HPV types (16, 18, 31, 33, 35, 39, 45, 51, 52, 56, 58, 59, 66, and 68) with specific identification of HPV-16 and HPV-18.

##### 2.4.1.2 Serum cytokines and survivin measurement

Serum concentrations of IL-17, TNF-α, IFN-γ, TGF-β, and survivin were quantified using commercial sandwich enzyme-linked immunosorbent assay (ELISA) kits (e.g., IL-17: Cat# D1700, R&D Systems, Minneapolis, MN, USA; TNF-α: Cat# DTA00D, R&D Systems; IFN-γ: Cat# DIF50, R&D Systems; TGF-β: Cat# DB100B, R&D Systems; Survivin: Cat# ab119892, Abcam, Cambridge, UK). All assays were performed in duplicate according to the respective manufacturer’s instructions. Absorbance was read using a microplate reader (Model: SpectraMax M5, Molecular Devices, San Jose, CA, USA).

##### 2.4.1.3 Immunoglobulin quantification

Serum levels of immunoglobulins A, G, and M (IgA, IgG, IgM) were measured by scattering immunoturbidimetry on an automated clinical chemistry analyzer (Model: AU5800, Beckman Coulter, Brea, CA, USA) using the manufacturer’s calibrated reagents.

##### 2.4.1.4 Immunohistochemistry (Ki-67)

Ki-67 protein expression in cervical tissue specimens was assessed by immunohistochemistry using the streptavidin-peroxidase (SP) method. Briefly, 4-μm formalin-fixed, paraffin-embedded tissue sections were deparaffinized, rehydrated, and subjected to antigen retrieval. Sections were then incubated with a mouse monoclonal anti-human Ki-67 primary antibody (Clone: MIB-1, Dako/Agilent, Santa Clara, CA, USA) at a dilution of 1:100. Detection was performed using a Dako EnVision+ System-HRP (DAB) kit. Ki-67 labeling index was calculated as the percentage of positively stained nuclei among at least 500 epithelial cells counted in representative high-power fields, evaluated independently by two experienced pathologists blinded to the clinical data.

### 2.5 Grouping and treatment regimens

Eligible patients were divided into two groups according to different local adjuvant interventions applied after LEEP. All patients underwent standardized LEEP at our hospital, and different local treatment strategies were selected postoperatively based on previous clinical medication regimens. Patients in the control group (n = 102) received interferon α-2b gel as local vaginal adjuvant therapy after completion of LEEP and postoperative recovery. The drug used was interferon α-2b gel (manufactured by Hefei Zhaoke Pharmaceutical Co., Ltd.; National Drug Approval No. S20020079). The specific administration schedule was as follows: medication was initiated on the third day after the end of the first postoperative menstruation; the gel was administered vaginally once every other day, with a dose of approximately 1–2 g each time. Ten administrations constituted one treatment course, and a total of two courses were completed. Patients in the observation group (n = 105) received anti-HPV gel as postoperative adjuvant therapy after LEEP. The preparation used was anti-HPV gel (Hunan Hualian Kang Biotechnology Co., Ltd.; Medical Device Registration No. Xiang Medical Device Approval Certificate 20202180436). The treatment regimen was as follows: use was initiated on the third day after the first postoperative menstruation had completely ended. After vulvar cleansing before bedtime, the anti-HPV gel applicator was inserted vaginally to the cervical area. The gel was used once every other day, one applicator per use. Ten applications constituted one course, and a total of two courses were administered. During the treatment period, patients in both groups received uniform routine postoperative care and health education, including guidance on vaginal hygiene, recommendations regarding sexual activity, and scheduled follow-up visits. To avoid the influence of confounding factors on the study outcomes, no other antiviral, immunomodulatory, or drugs potentially affecting the observation indicators were used during the treatment and follow-up periods.

### 2.6 Observation indicators

#### 2.6.1 Clinical efficacy evaluation

After completion of two courses of local adjuvant therapy, the clinical efficacy in both groups was comprehensively evaluated. Efficacy assessment was based on improvement in clinical symptoms, HPV infection status, and recovery of vaginal microecology. The criteria were defined as follows: Markedly effective: clinical symptoms and signs were significantly alleviated or almost completely resolved, HR-HPV testing converted to negative, and vaginal microecological indicators returned to the normal range. Effective: clinical symptoms and signs were improved compared with Before treatment, HPV test results remained stable or converted to negative, and vaginal microecological status was significantly improved but not fully normalized. Ineffective: clinical symptoms and signs showed no obvious improvement or remained unchanged, HPV test results showed no significant change, and vaginal microecological indicators showed no marked recovery. Total effective rate = (number of markedly effective cases + number of effective cases)/total number of cases × 100%. Overall clinical efficacy was treated as a composite endpoint; vaginal microecological indicators were additionally analyzed separately as secondary outcomes.

#### 2.6.2 Vaginal microecological status

After completion of two courses of treatment, vaginal secretion samples were collected. The vaginal secretion pH value was first measured, followed by microscopic observation and analysis of the vaginal microecological environment. Vaginal microecological recovery was evaluated comprehensively based on pH value, bacterial flora morphology, biochemical indicators, and the Nugent score (12), according to the following criteria: Microecological recovery to normal: vaginal secretion pH ≤ 4.5; Lactobacillus-dominant flora with diverse morphology and uniform distribution; bacterial density ≤ 5 per high-power field; both sialidase and hydrogen peroxide tests negative; Nugent score ≤ 3. Microecological non-recovery: vaginal secretion pH > 4.5; flora dominated by short bacilli or cocci with a marked reduction in Lactobacillus; bacterial density > 5 per high-power field; sialidase or hydrogen peroxide test positive; Nugent score > 3.

#### 2.6.3 Detection of inflammatory response indicators

Before treatment and After treatment, 5 mL of fasting venous blood was collected from patients in the early morning. After centrifugation at 3500 rpm for 10 min, the supernatant was collected. Serum levels of interleukin-17 (IL-17), tumor necrosis factor-α (TNF-α), interferon-γ (IFN-γ), and transforming growth factor-β (TGF-β) were measured using enzyme-linked immunosorbent assay (ELISA), strictly following the manufacturers’ instructions.

#### 2.6.4 Detection of immune function indicators

Before treatment and After treatment, the reserved serum samples obtained as described in Section 1.5.3 were used to measure serum immunoglobulin A (IgA), immunoglobulin G (IgG), and immunoglobulin M (IgM) levels by scattering immunoturbidimetry, to evaluate changes in humoral immune function.

#### 2.6.5 Detection of cell proliferation–related indicators

Stored fasting serum samples (Section 1.5.3) were used to measure serum survivin levels by ELISA. Ki-67 expression in cervical tissue was evaluated semi-quantitatively by immunohistochemistry (SP method) using surgical pathological specimens (baseline) and, when clinically indicated, follow-up cervical tissue/biopsy specimens obtained during follow-up (post-treatment). Post-treatment Ki-67 was analyzed in patients with available specimens; the number of evaluable cases is reported in the tables. Ki-67 was expressed as the percentage of Ki-67–positive cells.

#### 2.6.6 Safety and HPV recurrence

The occurrence of adverse reactions during treatment was recorded in both groups, including local irritation symptoms, vaginal bleeding, increased vaginal discharge, and rash. After completion of treatment, patients were followed up for 6 months to observe HPV recurrence and to compare recurrence rates between the two groups. The criterion for HPV recurrence (13) was defined as re-detection of HR-HPV (positive by the same HPV assay) at follow-up in patients who had previously converted to HPV-negative status.

#### 2.6.7 Diagnostic performance and clinical utility analysis (exploratory)

To further evaluate the clinical usefulness of post-treatment indicators for predicting HR-HPV recurrence during follow-up, an exploratory prediction model was planned/constructed using candidate predictors including treatment group, post-treatment vaginal microecological indices (e.g., pH, Nugent score, Lactobacillus dominance), inflammatory and immune markers, survivin, and (when available) Ki-67. Model discrimination was assessed using receiver operating characteristic (ROC) curves and the area under the curve (AUC) with 95% confidence intervals. Calibration was assessed using calibration plots and the Hosmer–Lemeshow goodness-of-fit test. Clinical utility was evaluated using decision curve analysis (DCA) by estimating net benefit across a range of clinically relevant threshold probabilities. Internal validation was performed using bootstrap resampling (e.g., 1000 iterations) when individual-level data were available.

### 2.7 Statistical methods

Statistical analysis was performed using GraphPad Prism 8 software (GraphPad Software, San Diego, CA, USA). Continuous variables were tested for normality using the Shapiro–Wilk test. Normally distributed data are presented as mean ± standard deviation (SD) and compared between groups using independent two-sample Student’s t tests. Non-normally distributed data are expressed as median with interquartile range (IQR) and analyzed using the Mann–Whitney *U* test. Categorical variables are summarized as numbers (percentages) and compared between groups using the chi-square (χ^2^) test or Fisher’s exact test, as appropriate. All tests were two-sided, and a *P* value < 0.05 was considered statistically significant.

To enhance clinical interpretability, unadjusted effect sizes with 95% confidence intervals (CIs) were calculated from summary data. For binary outcomes (e.g., total effective rate, HPV recurrence), risk ratios (RRs) and risk differences (RDs) were estimated; the number needed to treat (NNT) was derived for major beneficial outcomes. For continuous outcomes (e.g., serum survivin, tissue Ki-67, inflammatory markers), mean differences (MDs) and standardized effect sizes (Hedges’ g) were computed based on post-treatment group means and SDs.

An exploratory prediction model was constructed to evaluate the ability of post-treatment indicators to discriminate patients who experienced HR-HPV recurrence during follow-up. Candidate predictors included treatment group, post-treatment vaginal microecological indices (pH, Nugent score, lactobacillus dominance), inflammatory and immune markers, serum survivin, and tissue Ki-67 (when available). Model discrimination was assessed using receiver operating characteristic (ROC) curves and the area under the curve (AUC) with 95% CIs. Calibration was evaluated with calibration plots and the Hosmer–Lemeshow goodness-of-fit test. Clinical utility was examined using decision curve analysis (DCA) to estimate the net benefit across a range of clinically relevant threshold probabilities. Internal validation was performed using bootstrap resampling with 1000 iterations. All analyses were conducted on an available-case basis; no imputation was performed for missing data.

Given the retrospective, non-randomized nature of this study, the assignment of patients to the Anti-HPV gel or Interferon α-2b gel group was based on clinical practice rather than randomization. Furthermore, the investigators involved in data collection and outcome assessment (e.g., evaluation of clinical efficacy, vaginal microecology, and analysis of laboratory biomarkers from retrieved records) were not blinded to the treatment group allocation. These aspects of the design should be considered when interpreting the observed associations, as they may introduce potential bias.

## 3 Results

### 3.1 Comparison of baseline characteristics

A total of 207 patients with HSIL complicated by HR-HPV infection who met the inclusion criteria were enrolled in this study, including 102 patients in the control group and 105 patients in the observation group. The two groups showed similar distributions in general demographic characteristics such as age and body mass index (BMI), with no evident baseline imbalance. Regarding the spectrum of HR-HPV infection, both groups were predominantly infected with HPV-16 and HPV-18, while other high-risk genotypes, including HPV-31, HPV-33, HPV-52, and HPV-58, were also observed. No significant differences were found in the composition ratios of individual HR-HPV subtypes between the two groups. Further comparison of the number of co-infecting HPV subtypes revealed comparable proportions of single-subtype infection and multiple-subtype mixed infection in both groups. Overall, no statistically significant differences were observed between the two groups in baseline characteristics or HR-HPV infection profiles (P>0.05), indicating good comparability between groups (**Table 1**).

**Table 1:** Comparison of baseline characteristics.

Characteristic	Control group (n=102)	Observation group (n=105)	*P* value
Demographic data			
Age (years), mean ± SD	39.95 ± 7.64	41.32 ± 8.91	0.237
BMI (kg/m^2^), mean ± SD	23.61 ± 3.19	24.04 ± 3.27	0.339
Menstrual & reproductive history			
Menarche age (years), mean ± SD	13.2 ± 1.5	13.4 ± 1.6	0.372
Gravidity, median (IQR)	2 (1–3)	2 (1–3)	0.682
Parity, median (IQR)	1 (1–2)	1 (1–2)	0.612
HR-HPV genotype distribution†	n (%)	n (%)	0.523
HPV-16	46 (45.10%)	52 (49.52%)	
HPV-18	18 (17.65%)	15 (14.29%)	
HPV-31	9 (8.82%)	11 (10.48%)	
HPV-33	7 (6.86%)	6 (5.71%)	
HPV-52	13 (12.75%)	10 (9.52%)	
HPV-58	9 (8.82%)	11 (10.48%)	
Number of HR-HPV subtypes	n (%)	n (%)	0.506
Single subtype	61 (59.80%)	58 (55.24%)	
Two or more subtypes	41 (40.20%)	47 (44.76%)	
Cytology/histology before LEEP	n (%)	n (%)	0.542
ASC-H	18 (17.65%)	22 (20.95%)	
HSIL (CIN2)	54 (52.94%)	51 (48.57%)	
HSIL (CIN3)	30 (29.41%)	32 (30.48%)	
Vaginal microecology at baseline			
pH, mean ± SD	5.1 ± 0.6	5.2 ± 0.7	0.307
Nugent score, mean ± SD	6.8 ± 2.1	7.0 ± 2.3	0.502
Lactobacillus dominance, n (%)	28 (27.45%)	30 (28.57%)	0.854
Comorbidities & habits	n (%)	n (%)	0.64
Smoking (ever)	12 (11.76%)	10 (9.52%)	
Diabetes	8 (7.84%)	6 (5.71%)	
Chronic cervicitis	45 (44.12%)	48 (45.71%)	

† For patients infected with two or more HR-HPV subtypes, the predominant genotype reported by the assay (e.g., highest viral load or signal intensity) was recorded as the primary genotype for distribution analysis. Multiple infection status is presented separately under “Number of HR-HPV subtypes”.

Data are presented as mean ± SD, median (IQR), or n (%).

ASC-H = Atypical squamous cells, cannot exclude HSIL; CIN = cervical intraepithelial neoplasia.

### 3.2 Comparison of clinical efficacy

After completion of two treatment courses, the overall clinical efficacy of the two groups was comprehensively evaluated. The observation group demonstrated a higher proportion of markedly effective cases and a lower proportion of ineffective cases compared with the control group. Further analysis of overall efficacy showed that the total effective rate in the observation group was significantly higher than that in the control group, with a statistically significant difference between groups (P<0.05). The Anti-HPV gel group achieved a significantly higher total clinical effective rate than the Interferon α-2b gel group (87.62% vs. 75.49%, P = 0.024), indicating its superior efficacy as a postoperative adjuvant(**Table 2**).

**Table 2:** Comparison of clinical efficacy.

Clinical efficacy	Control group (n=102)	Observation group (n=105)	*x* ^2^	*P*
Markedly effective	36 (35.29%)	53 (50.48%)		
Effective	41 (40.20%)	39 (37.14%)		
Ineffective	25 (24.51%)	13 (12.38%)		
Total effective rate	77 (75.49%)	92 (87.62%)	5.078	0.024

### 3.3 Comparison of vaginal microecological status

Regarding vaginal microecological indicators, both groups showed varying degrees of improvement after treatment, with more pronounced improvements observed in the observation group. Specifically, vaginal secretion pH values in the observation group shifted toward the physiological range, Nugent scores decreased markedly, and the proportion of patients with restoration of normal vaginal microecology was significantly higher than that in the control group. Statistical analysis demonstrated that all these differences between groups were statistically significant (P<0.05). These results indicate that anti-HPV gel may be more effective in promoting vaginal microecological reconstruction and restoring vaginal environmental homeostasis after LEEP (**Table 3**).

**Table 3:** Comparison of vaginal microecological status.

Index	Control group (n=102)	Observation group (n=105)	*t/x^2^*	*P*
pH value	4.78 ± 0.42	4.29 ± 0.37	8.913	<0.001
Nugent score	4.52 ± 1.37	2.96 ± 1.15	8.883	<0.001
Vaginal microecology			6.013	0.014
Restored to normal	72 (70.59%)	89 (84.76%)		
Not restored	30 (29.41%)	16 (15.24%)		

### 3.4 Comparison of inflammatory response indicators

Before treatment, serum levels of IL-17, TNF-α, IFN-γ, and TGF-β were comparable between the two groups, with no statistically significant differences. After treatment, all these inflammatory markers decreased significantly in both groups, indicating that local adjuvant therapy helped alleviate systemic inflammatory responses. Notably, the magnitude of reduction in each inflammatory marker was significantly greater in the observation group than in the control group, with statistically significant intergroup differences (P<0.05). These findings suggest that combining anti-HPV gel with LEEP may more effectively inhibit inflammation-related pathway activation and improve both local and systemic inflammatory status (**Table 4**).

**Table 4:** Comparison of inflammatory response indicators (pg/mL).

Index	Control group (n=102)	Observation group (n=105)	*t*	*P*
IL-17				
Before treatment	38.65 ± 7.92	40.26 ± 8.53	1.406	0.161
After treatment	27.47 ± 6.25^a^	21.19 ± 5.87^a^	7.453	<0.001
TNF-α				
Before treatment	42.18 ± 9.36	43.72 ± 10.18	1.132	0.258
After treatment	30.62 ± 7.54^a^	24.36 ± 6.45^a^	6.425	<0.001
IFN-γ				
Before treatment	29.83 ± 6.47	30.59 ± 7.27	0.793	0.428
After treatment	21.91 ± 5.36^a^	17.26 ± 4.68^a^	6.654	<0.001
TGF-β				
Before treatment	58.49 ± 11.72	60.27 ± 12.53	1.054	0.292
After treatment	43.75 ± 9.64^a^	36.88 ± 8.95^a^	5.315	<0.001

^a^ indicates P<0.05 compared with Before treatment within the same group.

### 3.5 Comparison of immune function indicators

Immune function assessment showed no significant differences in IgA, IgG, or IgM levels between the two groups before treatment. After treatment, levels of all three immunoglobulins increased significantly in both groups, indicating improvement in immune function. Further comparison revealed that the increases in IgA, IgG, and IgM levels were significantly greater in the observation group than in the control group, with statistically significant differences (P<0.05). These results suggest that anti-HPV gel may play a more active role in enhancing immune responses and improving immune function (**Table 5**).

**Table 5:** Comparison of immune function indicators (g/L).

Index	Control group (n=102)	Observation group (n=105)	*t*	*P*
IgA				
Before treatment	1.62 ± 0.41	1.69 ± 0.47	1.14	0.255
After treatment	2.01 ± 0.52^a^	2.38 ± 0.56^a^	4.922	<0.001
IgG				
Before treatment	10.93 ± 2.14	11.31 ± 2.32	1.224	0.222
After treatment	13.25 ± 2.48^a^	15.19 ± 2.76^a^	5.314	<0.001
IgM				
Before treatment	1.07 ± 0.28	1.13 ± 0.31	1.46	0.145
After treatment	1.42 ± 0.33^a^	1.71 ± 0.38^a^	5.855	<0.001

^a^ indicates P<0.05 compared with Before treatment within the same group.

### 3.6 Comparison of cell proliferation-related indicators

Before treatment, both groups showed relatively high serum survivin levels and tissue Ki-67 expression, with no statistically significant intergroup differences. After treatment, both indicators decreased significantly in both groups, suggesting that adjuvant therapy could suppress abnormal cell proliferation to some extent. Importantly, the reductions in survivin levels and Ki-67 expression were significantly greater in the observation group than in the control group, with statistically significant differences (P<0.05). These findings indicate that anti-HPV gel, when used as adjuvant therapy after LEEP, may be more beneficial in inhibiting abnormal cell proliferation associated with lesion progression (**Table 6**).

**Table 6:** Comparison of cell proliferation-related indicators.

Index	Control group (n=102)	Observation group (n=105)	*t*	*P*
Survivin (ng/mL)				
Before treatment	6.42 ± 1.35	6.71 ± 1.48	1.471	0.142
After treatment	4.98 ± 1.12a	3.87 ± 0.96a	7.663	<0.001
Tissue Ki-67 (%)				
Before treatment	42.65 ± 9.18	43.83 ± 10.32	0.868	0.386
After treatment	31.43 ± 8.26a	24.67 ± 7.51a	6.164	<0.001

^a^ indicates P<0.05 compared with Before treatment within the same group. Post-treatment tissue Ki-67 was assessed in patients with available follow-up cervical biopsy specimens.

### 3.7 Comparison of adverse reactions and HPV recurrence

Safety analysis showed that both groups mainly experienced mild local adverse reactions during treatment, including local irritation, slight vaginal bleeding, and increased vaginal discharge. No statistically significant difference was observed in the overall incidence of adverse reactions between the two groups (P>0.05), indicating good overall tolerability of both adjuvant treatment regimens. During the 6-month follow-up period, the HPV recurrence rate in the observation group was significantly lower than that in the control group, with a statistically significant difference between groups (P<0.05). These results suggest that, while maintaining safety, anti-HPV gel may have a potential advantage in reducing short-term HPV recurrence risk (**Table 7**).

**Table 7:** Comparison of adverse reactions and HPV recurrence.

Adverse reactions	Control group (n=102)	Observation group (n=105)	*x^2^*	*P*
Local irritation symptoms	6 (5.88%)	4 (3.81%)		
Vaginal bleeding	4 (3.92%)	3 (2.86%)		
Increased discharge	3 (2.94%)	4 (3.81%)		
Rash	1 (0.98%)	0 (0.00%)		
Total incidence	14 (13.73%)	11 (10.48%)	0.514	0.473
6-month HPV recurrence rate	17 (16.67%)	7 (6.67%)	5.047	0.024

### 3.8 Effect size analysis of key outcomes

To enhance clinical interpretability, unadjusted effect sizes with 95% confidence intervals (CIs) were calculated from the summary data. For categorical outcomes, risk ratios (RRs) and risk differences (RDs) were calculated; the number needed to treat (NNT) is provided for major benefits. For continuous outcomes, mean differences (MDs) and standardized effect sizes (Hedges’ g) were calculated based on post-treatment values (**Table 8**).

**Table 8:** Effect size analysis of key outcomes (unadjusted).

Outcome	Control	Observation	Effect size (95% CI)	Additional metrics
Total effective rate	77/102 (75.49%)	92/105 (87.62%)	RR 1.16 (1.02, 1.32) RD 0.121 (0.017, 0.226)	NNT 8.2
Microecology restored	72/102 (70.59%)	89/105 (84.76%)	RR 1.20 (1.03, 1.39) RD 0.142 (0.030, 0.254)	NNT 7.1
6-month HPV recurrence	17/102 (16.67%)	7/105 (6.67%)	RR 0.40 (0.17, 0.92) RD -0.100 (-0.187, -0.013)	NNT 10.0
Any adverse reaction	14/102 (13.73%)	11/105 (10.48%)	RR 0.76 (0.36, 1.60) RD -0.032 (-0.121, 0.056)	—
Serum survivin (ng/mL, post)	4.98 ± 1.12	3.87 ± 0.96	MD -1.11 (-1.39, -0.83)	Hedges’ g -1.06
Tissue Ki-67 (%, post)	31.43 ± 8.26	24.67 ± 7.51	MD -6.76 (-8.91, -4.61)	Hedges’ g -0.85
IL-17 (pg/mL, post)	27.47 ± 6.25	21.19 ± 5.87	MD -6.28 (-7.93, -4.63)	Hedges’ g -1.03
IgG (g/L, post)	13.25 ± 2.48	15.19 ± 2.76	MD 1.94 (1.23, 2.65)	Hedges’ g 0.74

Effect sizes are unadjusted; because this was a retrospective study, these estimates reflect associations and may be influenced by residual confounding. Post-treatment tissue Ki-67 values were calculated based on cases with available follow-up cervical biopsy specimens.

### 3.9 Diagnostic performance and clinical utility

**Figure 2:**
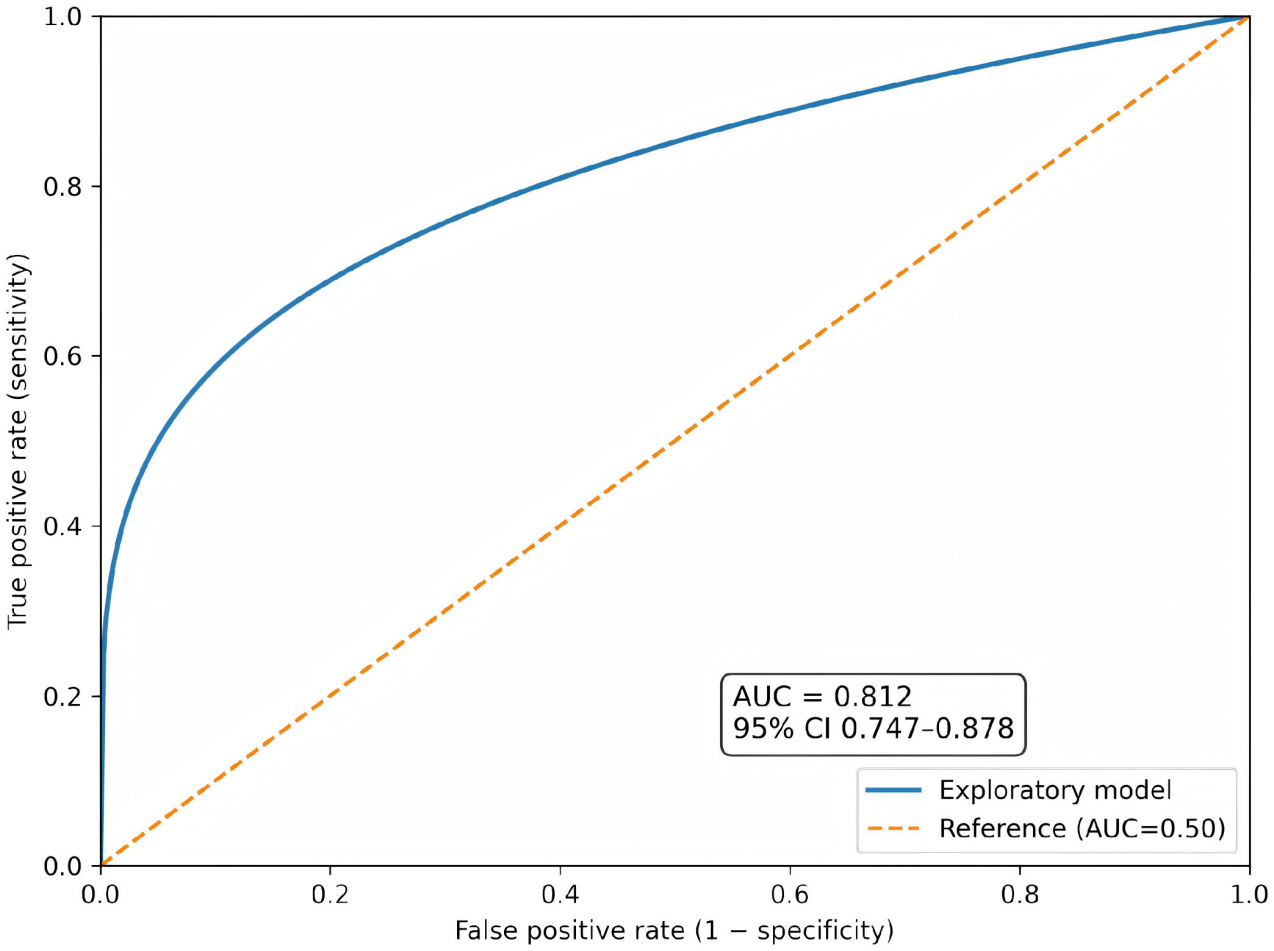
Receiver operating characteristic (ROC) curve of the exploratory model for predicting HR-HPV recurrence during follow-up.

**Figure 3:**
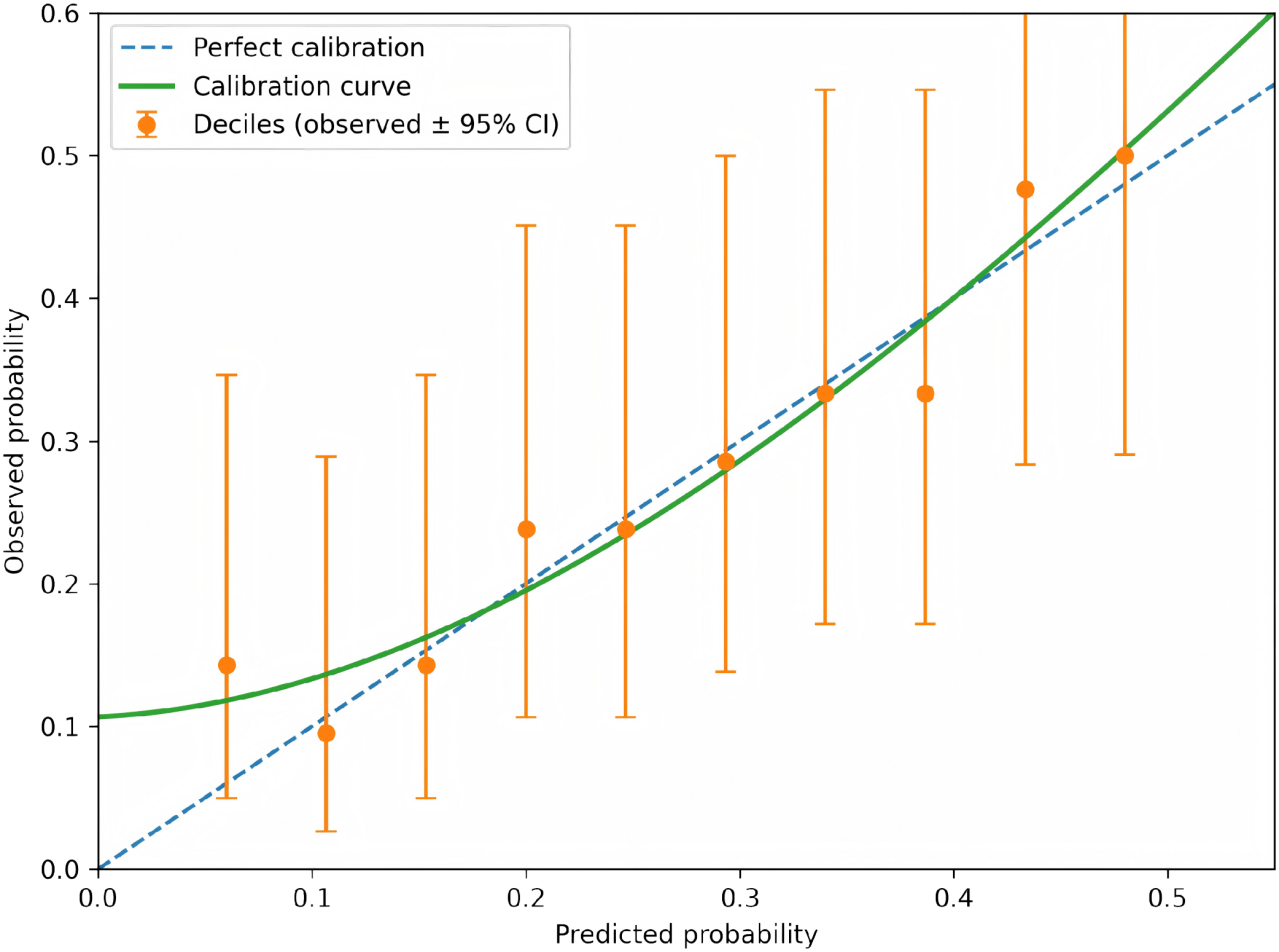
Calibration plot of the exploratory model for predicting HR-HPV recurrence during follow-up.

**Figure 4:**
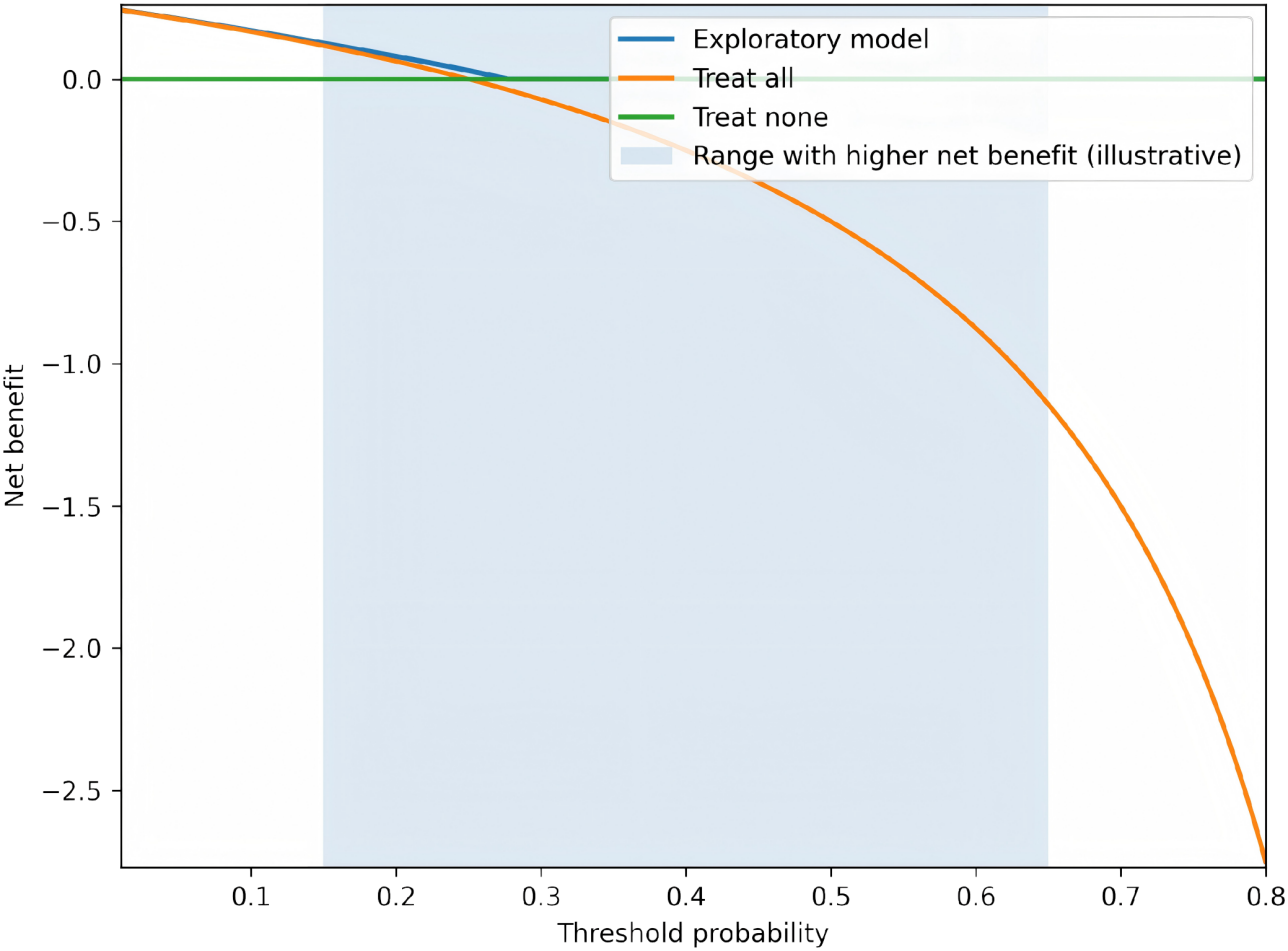
Decision curve analysis (DCA) showing net benefit of the exploratory model across a range of threshold probabilities.

To complement group-level comparisons, an exploratory prediction analysis was performed to assess whether post-treatment indicators could discriminate patients who experienced HR-HPV recurrence during follow-up. The ROC curve and corresponding AUC are shown in **Figure 2** (AUC = 0.812, 95% CI 0.747–0.878). Model calibration is presented in **Figure 3**, and decision curve analysis (DCA) is provided in **Figure 4** to illustrate potential clinical net benefit across threshold probabilities. These plots should be interpreted as exploratory and require confirmation with prospective data.

The model showed good calibration; predicted risks were closely aligned with observed probabilities across deciles.

The model demonstrated superior clinical net benefit compared with “treat all” and “treat none” strategies between threshold probabilities of 15–65%.

## 4 Discussion

HSIL is widely recognized as a critical precancerous stage in the development and progression of cervical cancer, and its natural course is highly dependent on the persistence of HR-HPV infection. Studies (14, 15) have shown that the long-term presence of HR-HPV is not only a necessary condition for lesion formation but also an important risk factor for postoperative residual disease and recurrence. Although LEEP can physically remove visible lesion tissue and achieve relatively favorable pathological clearance in the short term, its impact on potential viral reservoirs, local microenvironmental disruption, and immune imbalance is limited. Consequently, a certain proportion of patients remain at risk of persistent HPV infection and lesion recurrence after surgery (16). Therefore, exploring more effective adjuvant treatment strategies after LEEP to promote viral clearance and improve local ecological and immune status has become one of the key focuses in current clinical practice. Based on a real-world retrospective cohort, the present study comparatively analyzed the clinical effects of interferon α-2b gel and anti-HPV gel as postoperative adjuvant therapies following LEEP. The results showed that, in terms of overall clinical efficacy, the effective rate in the observation group was significantly higher than that in the control group, with a markedly lower proportion of ineffective cases. These findings suggest that, in the context of postoperative adjuvant intervention, traditional interferon regimens relying primarily on immune stimulation or immune enhancement mechanisms may be insufficient to adequately address the complex local virus–host microenvironment, whereas the comprehensive intervention advantages demonstrated by anti-HPV gel may be more consistent with the practical needs of postoperative HSIL management. Some previous clinical observations (17, 18) have also indicated that local therapeutic approaches targeting HPV infection that simultaneously address viral blockade and mucosal repair are often more conducive to improving short-term clearance rates and clinical outcomes. The results of the present study are largely consistent with these observations. In addition, vaginal microecological homeostasis plays an important regulatory role in the occurrence, persistence, and clearance of HR-HPV infection (19, 20). Under healthy conditions, a Lactobacillus-dominated vaginal microbiota maintains an acidic environment, inhibits pathogen colonization, and enhances mucosal barrier function, thereby providing fundamental support for local cervical immune defense. However, LEEP itself and prior viral infection can disrupt vaginal microecological structure, creating favorable conditions for HPV persistence. In this study, patients in the observation group showed a significant decrease in vaginal secretion pH values after treatment, a marked improvement in Nugent scores, and a significantly higher proportion of microecological restoration to normal compared with the control group. Zhang et al. (21) suggested that reconstruction of vaginal microecology not only helps reduce HPV viral load but may also indirectly influence lesion outcomes by modulating local inflammatory responses and immune reactions. The present findings further support the potential advantage of anti-HPV gel in promoting vaginal microecological remodeling, which may be attributable to its multiple actions, including the formation of a local protective barrier, reduction of pathogen re-invasion, and improvement of the mucosal repair environment.

Inflammatory responses are considered an important bridge linking persistent HPV infection with cervical lesion progression. Continuous viral stimulation can induce the release of local and systemic inflammatory cytokines, and chronic inflammation is not only unfavorable for viral clearance but may also promote abnormal cell proliferation through activation of multiple signaling pathways (22, 23). The results of this study showed that serum levels of IL-17, TNF-α, IFN-γ, and TGF-β in both groups were significantly reduced After treatment compared with Before treatment, indicating that postoperative adjuvant therapy overall contributed to alleviating inflammatory burden. Notably, the magnitude of reduction in inflammatory factors was more pronounced in the anti-HPV gel group. Previous studies (24, 25) have demonstrated that IL-17 and TNF-α play key roles in amplifying chronic cervical inflammation and tissue injury, while IFN-γ and TGF-β are critically involved in the formation of an immunosuppressive microenvironment. The present findings suggest that anti-HPV gel may attenuate inflammatory cascade reactions at the source by reducing persistent viral antigen stimulation, thereby achieving a more stable anti-inflammatory effect. This observation is consistent with findings from other studies investigating locally applied HPV-targeted therapies (26, 27). Immune function status is an important intrinsic factor determining the outcome of HR-HPV infection. HPV can evade immune surveillance through multiple mechanisms, leading to impaired local and systemic immune function and, consequently, persistent infection (28). In this study, levels of IgA, IgG, and IgM were significantly increased After treatment in both groups, indicating a certain degree of recovery in humoral immune function, with more pronounced improvements observed in the anti-HPV gel group. Relevant studies (29) have pointed out that improvements in immunoglobulin levels not only reflect enhanced antigen-response capacity but may also be closely related to reduced local infection burden and alleviation of immunosuppressive states. The results of this study suggest that the advantage of anti-HPV gel in improving immune function may not stem from direct immune stimulation, but rather from indirect restoration of immune homeostasis through improvement of the local microenvironment and promotion of viral clearance. This “de-burdening--recovery” mechanistic pattern appears to be reasonable in the management of chronic infections.

With respect to cell proliferation-related indicators, this study further explored the potential effects of different adjuvant treatment strategies at the molecular level. Survivin and Ki-67 are important biomarkers reflecting cell proliferative activity and apoptosis imbalance, and their high expression is often associated with cervical lesion progression and poor prognosis (30, 31). In this study, levels of these indicators decreased significantly After treatment in both groups, with a more pronounced reduction observed in the observation group. Previous studies (32, 33) have suggested that inflammatory microenvironments, immune evasion, and persistent viral stimulation can collectively drive the activation of abnormal cell proliferation signaling pathways. The observed superiority of anti-HPV gel in suppressing survivin and Ki-67 expression in this study may be related to the synergistic effects of its actions in reducing inflammation, improving immune status, and restoring microecological balance. This multimodal mechanism may collectively disrupt the critical pathways supporting persistent lesions and recurrence. Finally, in terms of safety and recurrence, the results of this study showed that the incidence of adverse reactions was comparable between the two groups and mainly consisted of mild local discomfort, with no obvious serious adverse events observed. This indicates that both treatment regimens have good tolerability in clinical application. Notably, the recurrence rate during the 6-month follow-up period was significantly lower in the observation group than in the control group. Previous studies (34) have shown that persistent HPV infection, microecological imbalance, and residual abnormal proliferative signaling are important drivers of postoperative recurrence. The observed difference in recurrence rates in this study may represent a comprehensive clinical manifestation of multiple biological improvements and further supports the potential value of anti-HPV gel as an adjuvant therapy after LEEP.

Beyond group-level comparisons, this study also conducted an exploratory analysis to evaluate whether post-treatment indicators could collectively predict the risk of HR-HPV recurrence during follow-up. The constructed model demonstrated good discriminatory ability, with an AUC of 0.812 (95% CI: 0.747–0.878), suggesting that a combination of clinical, microecological, inflammatory, immune, and proliferative biomarkers after adjuvant therapy may effectively stratify patients according to their recurrence risk. Furthermore, the model exhibited satisfactory calibration across risk deciles, and decision curve analysis indicated a positive net clinical benefit within a clinically relevant threshold probability range (15–65%). These findings imply that such a prediction tool could potentially assist in identifying high-risk patients who may require closer surveillance or intensified intervention after LEEP, thereby facilitating more personalized postoperative management. However, it must be emphasized that the current model is exploratory and derived from a retrospective single-center cohort; its generalizability and clinical utility need to be validated in prospective, multicenter studies with larger sample sizes.

### 4.1 Limitations and future perspectives

Although this study, conducted in a real-world clinical setting, relatively systematically compared the application effects of interferon α-2b gel and anti-HPV gel as adjuvant therapies after LEEP in patients with HSIL complicated by HR-HPV infection, and performed a comprehensive evaluation from multiple dimensions including clinical efficacy, vaginal microecology, inflammatory response, immune function, and cell proliferation--related indicators, several limitations should still be acknowledged objectively. (1) This study adopted a retrospective single-center design, with cases derived from the same medical institution. As a retrospective study without randomization or blinding, there is an inherent risk of selection bias and measurement bias, despite our efforts to balance baseline characteristics and use standardized data extraction. The selection of study participants was influenced to some extent by previous clinical diagnostic and treatment processes as well as the completeness of medical records, which may have introduced selection bias. Although the two groups were comparable in baseline characteristics, the potential influence of residual confounding factors on the study results cannot be completely excluded. Therefore, the observed associations between treatment type and outcomes should be interpreted as indicative rather than causal, given the non-randomized design. (2) The sample size of this study was relatively limited, and the statistical power was insufficient in some subgroup analyses and recurrence event evaluations. This limitation restricted the feasibility of more refined stratified analyses according to different HR-HPV subtypes, patterns of co-infection, or other potential influencing factors. Future studies with larger sample sizes and multicenter collaboration may help improve the robustness and generalizability of the findings. (3) The follow-up duration in this study was 6 months, focusing primarily on short-term efficacy and early recurrence, which is insufficient to comprehensively reflect the long-term impact of different adjuvant treatment regimens on HSIL recurrence risk and cervical cancer development. Given the long-term evolutionary nature of HPV infection and cervical lesions, extending the follow-up period and dynamically monitoring sustained viral clearance and lesion outcomes would contribute to a more thorough evaluation of long-term therapeutic benefits. (4) Although multiple inflammatory, immune, and cell proliferation--related indicators were assessed, these remained indirect biological markers, and in-depth exploration of the molecular mechanisms underlying the action pathways of anti-HPV gel was not performed. Future studies integrating histological, molecular biological, and multi-omics approaches may further elucidate the potential mechanisms from perspectives such as virus–host interactions and remodeling of the local immune microenvironment.

Based on these limitations, future research should employ multicenter, large-sample, prospective randomized controlled designs to validate the efficacy and safety of different adjuvant treatment strategies after LEEP with a higher level of evidence. Further clarification of the application value of anti-HPV gel across different populations, viral subtypes, and risk stratifications will provide a more solid evidence base for optimizing individualized postoperative management strategies for HSIL. Moreover, the exploratory prediction model developed in this study warrants external validation and refinement in independent cohorts. Future research could incorporate additional predictors such as viral load dynamics, host genetic factors, or novel immune markers to enhance predictive accuracy. Ultimately, the integration of effective adjuvant therapies with reliable risk-prediction tools may pave the way for a more stratified and precision-based approach to the management of HSIL after LEEP.

## 5 Conclusion

Compared with the combination of interferon α-2b gel, the addition of anti-HPV gel to LEEP treatment further improves the overall clinical response rate, significantly optimizes the vaginal microecological environment, alleviates inflammatory responses, enhances humoral immune function, and demonstrates certain advantages in inhibiting the expression of cell abnormal proliferation--related markers (survivin and Ki-67). Meanwhile, both adjuvant treatment regimens showed good tolerability in terms of safety, and anti-HPV gel exhibited a lower recurrence risk during short-term follow-up, suggesting its potential clinical value as an adjuvant therapy after LEEP. The exploratory prediction model based on post-treatment indicators also showed promising discriminative ability and clinical utility for identifying patients at higher risk of recurrence, which may support more individualized postoperative surveillance strategies. Overall, anti-HPV gel may provide a postoperative adjuvant intervention option with certain advantages for patients with HSIL complicated by HR-HPV infection through multi-level and multi-pathway regulatory effects. It should be emphasized that, as this study was a single-center retrospective analysis, the above conclusions still require further validation in higher-quality prospective studies. Future well-designed randomized controlled trials are expected to provide clearer and more reliable evidence for optimizing adjuvant treatment strategies after LEEP.

## Data Availability

The raw data supporting the conclusions of this article will be made available by the authors, without undue reservation.

## References

[1] Torous VF . Challenging lesions in cervical cytology: The elusive HSIL. Cytopathology. (2024) 35:48–59. doi: 10.1111/cyt.13303, PMID: 37706620

[2] Xia W Dai X Hu Y Yang S Chen C Li X . Value of intraoperative post-conisation human papillomavirus testing in predicting residual or recurrence after treatment with a loop electrosurgical excision procedure in women with HR-HPV positive and cervical high-grade squamous intraepithelial lesion. BMC Cancer. (2024) 24:1496. doi: 10.1186/s12885-024-13272-9, PMID: 39639227 PMC11619614

[3] Kim M Park NJ Jeong JY Park JY . Multiple human papilloma virus (HPV) infections are associated with HSIL and persistent HPV infection status in korean patients. Viruses. (2021) 13:1342. doi: 10.3390/v13071342, PMID: 34372548 PMC8310096

[4] Xie H Huang J Ye M Xu J Zou R Zhang Z . Integrated HPV testing, TCT, and colposcopic VIA/VILI improves detection rate for HSIL and cervical cancer: A retrospective cohort in China. Int J Womens Health. (2025) 17:4649–60. doi: 10.2147/IJWH.S551316, PMID: 41293762 PMC12641184

[5] Huang G Gao H Chen Y Lin W Shen J Xu S . Platelet-to-lymphocyte ratio (PLR) as the prognostic factor for recurrence/residual disease in HSIL patients after LEEP. J Inflammation Res. (2023) 16:1923–36. doi: 10.2147/JIR.S406082, PMID: 37152868 PMC10162391

[6] Fonseca FV de Carvalho NS Maestri CA Martins MF Kowacs DP . The role of HIV as an independent risk factor to cervical HSIL recurrence. Rev Bras Ginecol Obstet. (2024) 46:e-rbgo85. doi: 10.61622/rbgo/2024rbgo85, PMID: 39530067 PMC11554331

[7] Melani C Dowdell K Pittaluga S Dunleavy K Roschewski M Song JY . Interferon alfa-2b in patients with low-grade lymphomatoid granulomatosis and chemotherapy with DA-EPOCH-R in patients with high-grade lymphomatoid granulomatosis: an open-label, single-centre, phase 2 trial. Lancet Haematol. (2023) 10:e346–58. doi: 10.1016/S2352-3026(23)00029-7, PMID: 37011643

[8] Shi HJ Song H Zhao QY Tao CX Liu M Zhu QQ . Efficacy and safety of combined high-dose interferon and red light therapy for the treatment of human papillomavirus and associated vaginitis and cervicitis: A prospective and randomized clinical study. Med (Baltimore). (2018) 97:e12398. doi: 10.1097/MD.0000000000012398, PMID: 30213012 PMC6156011

[9] Inga P Pavel T Tatiana D Svetlana S Timur S Irina A . Interferon alpha-2b treatment for exophytic nasal papillomas and human papillomavirus infection. Braz J Otorhinolaryngol. (2024) 90:101449. doi: 10.1016/j.bjorl.2024.101449, PMID: 38972285 PMC11263936

[10] Hu Y Li M Liu J Huang Q Chen J Chen L . Anal canal condyloma acuminatum treated with anti-HPV biological dressing: clinical analysis of 64 cases. Indian J Dermatol. (2022) 67:204. doi: 10.4103/ijd.ijd_930_21, PMID: 36092239 PMC9455093

[11] Wang DY Cui YY Zhang WW Fan MS Qiu KX Yan L . Effect of different interventions on the treatment of high-risk human papillomavirus infection: a systematic review and network meta-analysis. Front Med (Lausanne). (2024) 11:1274568. doi: 10.3389/fmed.2024.1274568, PMID: 38420364 PMC10899477

[12] Lokken EM Jisuvei C Oyaro B Shafi J Nyaigero M Kinuthia J . Nugent score, amsel’s criteria, and a point-of-care rapid test for diagnosis of bacterial vaginosis: performance in a cohort of Kenyan women. Sex Transm Dis. (2022) 49:e22–5. doi: 10.1097/OLQ.0000000000001469, PMID: 33993164 PMC8590706

[13] Kechagias KS Kalliala I Bowden SJ Athanasiou A Paraskevaidi M Paraskevaidis E . Role of human papillomavirus (HPV) vaccination on HPV infection and recurrence of HPV related disease after local surgical treatment: systematic review and meta-analysis. Bmj. (2022) 378:e070135. doi: 10.1136/bmj-2022-070135, PMID: 35922074 PMC9347010

[14] Wang Z Gu Y Wang H Chen J Zheng Y Cui B . Distribution of cervical lesions in high-risk HPV (hr-HPV) positive women with ASC-US: a retrospective single-center study in China. Virol J. (2020) 17:185. doi: 10.1186/s12985-020-01455-2, PMID: 33228715 PMC7685609

[15] Chen L Dong B Zhang Q Mao X Lin W Ruan G . HR-HPV viral load quality detection provide more accurate prediction for residual lesions after treatment: a prospective cohort study in patients with high-grade squamous lesions or worse. Med Oncol. (2020) 37:37. doi: 10.1007/s12032-020-01363-z, PMID: 32232578

[16] Qin Y Li Q Ke X Zhang Y Shen X Wang W . Clearance of HR-HPV within one year after focused ultrasound or loop electrosurgical excision procedure in patients with HSIL under 30. Int J Hyperthermia. (2022) 39:15–21. doi: 10.1080/02656736.2021.2010817, PMID: 34937489

[17] Bénéteau T Groc S Murall CL Boué V Elie B Tessandier N . Incidence and duration of human papillomavirus infections in young women: insights from a bimonthly follow-up cohort. Infect Dis (Lond). (2025) 57:322–31. doi: 10.1080/23744235.2024.2427223, PMID: 39608970

[18] El-Zein M Ramanakumar AV Naud P Roteli-Martins CM de Carvalho NS Colares de Borba P . Determinants of acquisition and clearance of human papillomavirus infection in previously unexposed young women. Sex Transm Dis. (2019) 46:663–9. doi: 10.1097/OLQ.0000000000001053, PMID: 31464859 PMC6887628

[19] Huang J Yin C Wang J . Relationship between vaginal microecological changes and oncogene E6/E7 and high-risk human papillomavirus infection. J Obstet Gynaecol. (2023) 43:2161349. doi: 10.1080/01443615.2022.2161349, PMID: 36645341

[20] Wei B Chen Y Lu T Cao W Tang Z Yang H . Correlation between vaginal microbiota and different progression stages of cervical cancer. Genet Mol Biol. (2022) 45:e20200450. doi: 10.1590/1678-4685-gmb-2020-0450, PMID: 35320337 PMC8967114

[21] Zhang Z Yang Y Zhang L Wu Y Jia P Ma Q . Relationship between cervicovaginal microecological changes and HPV16/18 infection and cervical cancer in women of childbearing age. Ann Clin Lab Sci. (2023) 53:825–34. 38182150

[22] Ebrahimi F Rasizadeh R Jafari S Baghi HB . Prevalence of HPV in anal cancer: exploring the role of infection and inflammation. Infect Agent Cancer. (2024) 19:63. doi: 10.1186/s13027-024-00624-0, PMID: 39696546 PMC11654204

[23] Zhang Y Ma S He Z . Association between female HPV infection, inflammatory markers and depression: a cross-sectional and mediation analysis. BMC Infect Dis. (2025) 25:1329. doi: 10.1186/s12879-025-11590-2, PMID: 41094669 PMC12523160

[24] Akolkar K Sonar S Rao A Mamulwar M Ghule U Bagul R . Unraveling cervical inflammation in HIV-infected women: The regulatory role of miR-204-5p and miR-3691-3p. Life Sci. (2025) 377:123774. doi: 10.1016/j.lfs.2025.123774, PMID: 40449875

[25] Gauthier T Chen W . IFN-γ and TGF-β, crucial players in immune responses: A tribute to howard young. J Interferon Cytokine Res. (2022) 42:643–54. doi: 10.1089/jir.2022.0132, PMID: 36516375 PMC9917322

[26] Major AL Dvořák V Schwarzová J Skřivánek A Malík T Pluta M . Correction to: Efficacy and safety of an adsorbent and anti-oxidative vaginal gel on CIN1 and 2, on high-risk HPV, and on p16/Ki-67: a randomized controlled trial. Arch Gynecol Obstet. (2021) 303:513–4. doi: 10.1007/s00404-020-05925-4, PMID: 33415439 PMC8025267

[27] Guo X Qiu L Wang Y Wang Y Wang Q Song L . A randomized open-label clinical trial of an anti-HPV biological dressing (JB01-BD) administered intravaginally to treat high-risk HPV infection. Microbes Infect. (2016) 18:148–52. doi: 10.1016/j.micinf.2015.10.004, PMID: 26506570

[28] Shiri Aghbash P Rasizadeh R Sadri Nahand J Bannazadeh Baghi H . The role of immune cells and inflammasomes in Modulating cytokine responses in HPV-Related cervical cancer. Int Immunopharmacol. (2025) 145:113625. doi: 10.1016/j.intimp.2024.113625, PMID: 39637578

[29] Zheng JJ Miao JR Wu Q Yu CX Mu L . Correlation between HPV-negative cervical lesions and cervical microenvironment. Taiwan J Obstet Gynecol. (2020) 59:855–61. doi: 10.1016/j.tjog.2020.08.002, PMID: 33218401

[30] Chuwa AH Mvunta DH . Prognostic and clinicopathological significance of survivin in gynecological cancer. Oncol Rev. (2024) 18:1444008. doi: 10.3389/or.2024.1444008, PMID: 39687493 PMC11646728

[31] Voidăzan ST Dianzani C Husariu MA Geréd B Turdean SG Uzun CC . The Role of p16/Ki-67 Immunostaining, hTERC Amplification and Fibronectin in Predicting Cervical Cancer Progression: A Systematic Review. Biol (Basel). (2022) 11:956. doi: 10.3390/biology11070956, PMID: 36101337 PMC9312145

[32] Li R Weng X Hu X Wang J Zheng L . Pigment epithelium−derived factor inhibits proliferation, invasion and angiogenesis, and induces ferroptosis of extravillous trophoblasts by targeting Wnt−β−catenin/VEGF signaling in placenta accreta spectrum. Mol Med Rep. (2024) 29:75. doi: 10.3892/mmr.2024.13199, PMID: 38488028 PMC10975022

[33] Zhang Y Plansinis M Peak S Weber E Wei A Xu Y . Activation of toll-like receptor 2 promotes the expression of inflammatory mediators and cell proliferation of human polycystic kidney disease cells. Cell Signal. (2025) 131:111749. doi: 10.1016/j.cellsig.2025.111749, PMID: 40101851 PMC11994280

[34] Zang L Hu Y . Risk factors associated with HPV persistence after conization in high-grade squamous intraepithelial lesion. Arch Gynecol Obstet. (2021) 304:1409–16. doi: 10.1007/s00404-021-06217-1, PMID: 34482445

